# Book Review: Neuroscience for Psychologists: An Introduction

**DOI:** 10.3389/fpsyg.2021.737931

**Published:** 2021-08-24

**Authors:** Alejandra Rossi, Francisco J. Parada

**Affiliations:** Centro de Estudios en Neurociencia Humana y Neuropsicología, Facultad de Psicología, Universidad Diego Portales, Santiago, Chile

**Keywords:** psychology - study and teaching, neuroscience (psychology), 4E cognition, introduction, student resources

## Summary and Evaluation of the Book's Contents

The volume published by Springer Nature Switzerland and edited by Zeise (2021) is concerned with introducing Psychologists in particular -and social scientists in general- to Neuroscience. Two of the key insights that run through the 10 chapters written by scientists from Chile, Germany, and the USA are (i) the fact that neuroscience has come such a long way -in a relatively short period of time- that *newcomers will feel overwhelmed*; and (ii) that neuroscience is a transdiscipline, so it doesn't matter from which disciplinary corner you are coming from, *newcomers will be welcomed*.

The book starts very intimately, providing plenty of material for reflections. Dr. Zeise narrates the origins and motivations for editing the volume. He provides his vision on the role of a twenty-first-century psychologist -as both scientists and practitioners- providing the necessary framing for what is to come in the following chapters. The introduction leads the way to three “basic” science chapters. Central concepts of Biological and Life Sciences are revisited in “*Electrical Signals in the Nervous System*,” “*Basics of Neuropharmacology*,” and “*The Transmitters*.” This section basically provides a state-of-the-art brief on *The war of the soups and sparks* (Valenstein, 2006) and its aftermath.

The second section of the book provides a multidisciplinary perspective on Neuroscience, reviewing subfields of “special interest” for psychologists. Chapter 5 (“*Clinical Neuropharmacology*”) probably provides one of the best and most concise reviews on the topic. Newcomers and pre-med students alike will find the jam-packed chapter very useful for studying. Chapters 6 and 7 (“*Inputs, Outputs, and Multisensory Processing*” and “*Neuroplasticity in Humans*”) provide all necessary materials for a neuroscience-led critical discussion of one of the most characteristic debates in Psychology: *nature versus nurture*. Finally, the remaining three chapters are great introductions to advanced topics in Cognitive Neuroscience. Dr. Patricio Orio writes a concise and complete overview of “*Mathematical Modeling in Neuroscience*.” This chapter can become a quick-guide to relevant terms and concepts, including *Graph Theory* and *Network Neuroscience* (Sporns et al., 2005). Chapter 9 (“*Subjective Experience and Its Neural Basis*”) is an overview of subjectivity; probably the pinnacle of complexity in Psychology. Instead of reducing the topic or getting lost in the complexity, Dr. Ryan Smith achieves grounding evidence through associating generative models, which can accommodate a diverse range of epistemologies (Owens et al., 2018; Smith et al., 2019). The last chapter, “*Tools of Neuroscience*” is a concise chapter that provides a brief introduction to a plethora of imaging methods available for studying brain structure and function of both human and non-human animals, from invasive to non-invasive.

## Discussion of the Book's Content in Light of the Current Needs of the Community

*Neuroscience for Psychologists: An Introduction* provides students and researchers alike with a complete and concise state-of-the-art overview of Neuroscience, requiring almost no previous training on physics, biology, or mathematics. This is a much needed introductory volume, because Neuroscience helped shape modern Psychology; the ghost of foundational psychological dualism is nowadays almost completely exorcized. We must nevertheless point to the fact that Neuroscience has also, inadvertently, created a *dualist materialism*, which we will also overcome [see Mudrik and Maoz (2015) for a perspective]. Thus, further marrying Neuroscience to Psychology will facilitate the future development of transdisciplinary efforts based on complexity (Parada and Rossi, 2020).

Recent theoretical developments conceptualized the mind as an *Embodied, Embedded*, and *Extended* phenomenon, better understood from the *Enactive* approach [i.e., 4E-Cognition, see Newen et al. (2018)]. Neuroscience-inspired Psychologists can help humanity at large to focus resources and efforts on understanding *organic* and *artificial* cognitive abilities (Haselager and Gonzalez, 2007; Clark, 2008), forever forgetting *supernatural* accounts. Future psychologists, researchers and practitioners alike, will stand firmly with the idea that mental and physical health are one. Furthermore, since neuroscience is a biogenic approach (Lyon, 2006), cognitive abilities are understood as part of the complex phylogenetic continuum of planetary evolution (Allen and Bekoff, 1999; Margulis, 2008; Lyon et al., 2021). The 4E-Cognition perspective understands human health and well-being as complex phenomena product of people's multilevel interactions embedded in their social, cultural, and political environments.

Considering such paradigmatic changes in Neural and Cognitive Sciences, facts such as access to Education and Technology, Social Justice, and Planetary Health become primordial requirements for human well-being. We know these human ideals are implicit at the heart of *Neuroscience for Psychologists: An Introduction*. Nevertheless, considering the intimate tone of the introductory chapter and the current times we live in, it would have been great to read them explicitly. Likewise, given it is an introductory book -published in 2021- we would have hoped to see women scientists among the author list. Unfortunately, this is not the case. Perhaps evidencing an ages-long problem in (Neuro)Science (Diamond, 1963; Zippel, 2017; Machlovi et al., 2021), which Psychology and Social Sciences can help us resolve.

Finally, considering both the relevance for Psychology and how many resources already exist for -say Single-Cell Extracellular Recordings- we think the absence of *Mobile Brain/Body Imaging* (MoBI) in the present volume is critical. The last decade has seen an incredible revolution in mobile technologies for studying cognition in different scenarios other than our laboratories (Makeig et al., 2009; Gramann et al., 2014; Ladouce et al., 2016; Parada and Rossi, 2020). These technologies promise to revolutionize clinical research and practice (Johnson and Picard, 2020; King and Parada, 2021), launching the novel subfield of Real-World Neuroscience (Shamay-Tsoory and Mendelsohn, 2019). Psychological and brain sciences -in both concepts and curricula- should really be aware of these developments as they imply revolutionary consequences for both research and practice of Psychology.

In summary, this is a complete and concise volume. A motivated reader will find it easy to go through the pages as every chapter accomplishes their goals. The scope is adequate and will provide an excellent introduction to any psychologist and/or newcomer to Neuroscience, regardless of their background. Neuroscience is a transdisciplinary effort, and *Neuroscience for Psychologists: An Introduction* will precisely do that: provide a head start to anybody who is interested in walking the beautiful and challenging path to understanding the biophysics of human experience.

## Author Contributions

FP conceptualized the review. AR and FP wrote and edited the current manuscript for publication. All authors contributed to the article and approved the submitted version.

## Conflict of Interest

The authors declare that the research was conducted in the absence of any commercial or financial relationships that could be construed as a potential conflict of interest.

## Publisher's Note

All claims expressed in this article are solely those of the authors and do not necessarily represent those of their affiliated organizations, or those of the publisher, the editors and the reviewers. Any product that may be evaluated in this article, or claim that may be made by its manufacturer, is not guaranteed or endorsed by the publisher.

## References

[B1] Allen C. Bekoff M. (1999). Species of Mind: The Philosophy and Biology of Cognitive Ethology. MIT Press. Available online at: https://play.google.com/store/books/details?id=H97oavF8NO4C (accessed July 5, 2021).

[B2] Clark A. (2008). Supersizing the Mind: Embodiment, Action, and Cognitive Extension. Oxford: Oxford University Press. Available online at: https://play.google.com/store/books/details?id=n5wRDAAAQBAJ (accessed July 5, 2021). 10.1093/acprof:oso/9780195333213.001.0001

[B3] Diamond M. C. (1963). Women in modern science. J. Am. Med. Womens. Assoc. 18, 891–896. Available online at: https://www.ncbi.nlm.nih.gov/pubmed/14086067 (accessed July 5, 2021). 14086067

[B4] Gramann K. Ferris D. P. Gwin J. Makeig S. (2014). Imaging natural cognition in action. Int. J. Psychophysiol. 91, 22–29. 10.1016/j.ijpsycho.2013.09.003 24076470 PMC3983402

[B5] Haselager W. Gonzalez M. (2007). The meaningful body: on the differences between artificial and organic creatures, in Artificial Cognition Systems, eds Loula A. Gudwin R. Queiroz J. (IGI Global), 238–251. 10.4018/978-1-59904-111-7.ch008

[B6] Johnson K. T. Picard R. W. (2020). Advancing neuroscience through wearable devices. Neuron. 108, 8–12. 10.1016/j.neuron.2020.09.030 33058768

[B7] King J. L. Parada F. J. (2021). Using mobile brain/body imaging to advance research in arts, health, and related therapeutics. Eur. J. Neurosci. 10.1111/ejn.15313 33999462 PMC9291922

[B8] Ladouce S. Donaldson D. I. Dudchenko P. A. Ietswaart M. (2016). Understanding minds in real-world environments: toward a mobile cognition approach. Front. Hum. Neurosci. 10:694. 10.3389/fnhum.2016.00694 28127283 PMC5226959

[B9] Lyon P. (2006). The biogenic approach to cognition. Cogn. Process. 7, 11–29. 10.1007/s10339-005-0016-8 16628463

[B10] Lyon P. Keijzer F. Arendt D. Levin M. (2021). Reframing cognition: getting down to biological basics. Philos. Trans. R. Soc. Lond. B Biol. Sci. 376:20190750. 10.1098/rstb.2019.0750 33487107 PMC7935032

[B11] Machlovi S. Pero A. Ng S. Zhong M. Cai D. (2021). Women in neuroscience: where are we in 2019? J. Neurosci. Res. 99, 9–12. 10.1002/jnr.24570 32267025 PMC7754118

[B12] Makeig S. Gramann K. Jung T.-P. Sejnowski T. J. Poizner H. (2009). Linking brain mind and behavior. Int. J. Psychophysiol. 73, 95–100. 10.1016/j.ijpsycho.2008.11.008 19414039 PMC2796545

[B13] Margulis L. (2008). Symbiotic Planet: A New Look at Evolution. New York, NY: Basic Books.

[B14] Mudrik L. Maoz U. (2015). “Me & my brain”: exposing neuroscience's closet dualism. J. Cogn. Neurosci. 27, 211–221. 10.1162/jocn_a_00723 25244112

[B15] Newen A. De Bruin L. Gallagher S. (2018). The Oxford Handbook of 4E Cognition. London: Oxford University Press. 10.1093/oxfordhb/9780198735410.001.0001

[B16] Owens A. P. Allen M. Ondobaka S. Friston K. J. (2018). Interoceptive inference: from computational neuroscience to clinic. Neurosci. Biobehav. Rev. 90, 174–183. 10.1016/j.neubiorev.2018.04.017 29694845

[B17] Parada F. J. Rossi A. (2020). Perfect timing: mobile brain/body imaging scaffolds the 4E-cognition research program. Eur. J. Neurosci. 2020:14783. 10.1111/ejn.14783 32422692

[B18] Shamay-Tsoory S. G. Mendelsohn A. (2019). Real-life neuroscience: an ecological approach to brain and behavior research. Perspect. Psychol. Sci. 14, 841–859. 10.1177/1745691619856350 31408614

[B19] Smith R. Lane R. D. Parr T. Friston K. J. (2019). Neurocomputational mechanisms underlying emotional awareness: insights afforded by deep active inference and their potential clinical relevance. Neurosci. Biobehav. Rev. 107, 473–491. 10.1016/j.neubiorev.2019.09.002 31518636

[B20] Sporns O. Tononi G. Kötter R. (2005). The human connectome: a structural description of the human brain. PLoS Comput. Biol. 1:e42. 10.1371/journal.pcbi.0010042 16201007 PMC1239902

[B21] Valenstein E. S. (2006). The War of the Soups and the Sparks: The Discovery of Neurotransmitters and the Dispute Over How Nerves Communicate. New York, NY: Columbia University Press. 10.7312/vale13588

[B22] Zeise M. L. (2021). Neuroscience for psychologists: an introduction, in Introduction. ed Zeise M. L. (Cham: Springer Nature Switzerland). 1–10. 10.1007/978-3-030-47645-8

[B23] Zippel K. (2017). Women in Global Science: Advancing Academic Careers Through International Collaboration. Palo Alto, CA: Stanford University Press. 10.1515/9781503601505

